# CRHR1 mediates the transcriptional expression of pituitary hormones and their receptors under hypoxia

**DOI:** 10.3389/fendo.2022.893238

**Published:** 2022-09-02

**Authors:** Tong Ying Wang, Fang Yuan Xia, Jing Wen Gong, Xiao Kang Xu, Min Chao Lv, Mahanand Chatoo, Bilal Haider Shamsi, Meng Chen Zhang, Qian Ru Liu, Tian Xing Liu, Dan Dan Zhang, Xin Jiang Lu, Yang Zhao, Ji Zeng Du, Xue Qun Chen

**Affiliations:** ^1^ Department of Neurobiology, Department of Neurology of the Second Affiliated Hospital, Zhejiang University School of Medicine, Hangzhou, China; ^2^ National Health Commission and Chinese Academy of Medical Sciences Key Laboratory of Medical Neurobiology, Ministry of Education Frontier Science Center for Brain Research and Brain Machine Integration, School of Brain Science and Brain Medicine, Zhejiang University, Hangzhou, China; ^3^ Department of Research and Development, Jiuyuan Gene Engineering, Hangzhou, China; ^4^ Department of Pathology, and Department of Medical Oncology of the Second Affiliated Hospital, Zhejiang University School of Medicine, Hangzhou, China; ^5^ Department of Orthopedics, The Quzhou Affiliated Hospital of Wenzhou Medical University, Quzhou People’s Hospital, Quzhou, China; ^6^ Department of Cell and System Biology, University of Toronto, St. George, NB, Canada; ^7^ Department of Physiology, Zhejiang University School of Medicine, Hangzhou, China

**Keywords:** hypothalamus, hypoxia, transcription, pituitary, receptor, stress

## Abstract

Hypothalamus-pituitary-adrenal (HPA) axis plays critical roles in stress responses under challenging conditions such as hypoxia, *via* regulating gene expression and integrating activities of hypothalamus-pituitary-targets cells. However, the transcriptional regulatory mechanisms and signaling pathways of hypoxic stress in the pituitary remain to be defined. Here, we report that hypoxia induced dynamic changes in the transcription factors, hormones, and their receptors in the adult rat pituitary. Hypoxia-inducible factors (HIFs), oxidative phosphorylation, and cAMP signaling pathways were all differentially enriched in genes induced by hypoxic stress. In the pituitary gene network, hypoxia activated c-Fos and HIFs with specific pituitary transcription factors (*Prop1)*, targeting the promoters of hormones and their receptors. HIF and its related signaling pathways can be a promising biomarker during acute or constant hypoxia. Hypoxia stimulated the transcription of marker genes for microglia, chemokines, and cytokine receptors of the inflammatory response. Corticotropin-releasing hormone receptor 1 (CRHR1) mediated the transcription of *Pomc*, *Sstr2*, and *Hif2a*, and regulated the function of HPA axis. Together with HIF, c-Fos initiated and modulated dynamic changes in the transcription of hormones and their receptors. The receptors were also implicated in the regulation of functions of target cells in the pituitary network under hypoxic stress. CRHR1 played an integrative role in the hypothalamus-pituitary-target axes. This study provides new evidence for CRHR1 involved changes of hormones, receptors, signaling molecules and pathways in the pituitary induced by hypoxia.

## Introduction

Hypoxic stress has become common challenge to human and animals, which can induce enormous changes of gene expression for animal to maintain homeostasis in response to the varying levels of oxygen. Classically, HIFs are considered as main oxygen-sensors of homeostasis in physiological or pathophysiological processes. We and others found that CRHR1 is the major sub-type of CRHR receptors expressed in the corticotrophs, which plays critical but distinctive role in the activities of hypothalamus-pituitary-adrenal (HPA) axis in the pituitary. Evidence is accumulating that those changes of CRHR1 protein or transcript are important not only in the maintenance of homeostasis but also in the response to stress (1, 2).

Anterior pituitary gland contains five highly-differentiated cell types: 1) corticotrophs, which produce adrenocorticotrophic hormone (ACTH); 2) thyrotrophs, which produce thyroid stimulating hormone (TSH); 3) somatotrophs, which produce growth hormone (GH); 4) lactotrophs, which produce prolactin (PRL); and 5) gonadotrophs, which produce both follicle-stimulating hormone (FSH) and luteinizing hormone (LH). In the intermediate lobe, melanotrophs produce pro-opiomelanocortin (POMC), the precursor protein for corticotropin, melanocyte-stimulating hormone, and endorphin (3–6). In addition to these endocrine cells, non-endocrine cells can be also distinguished by numerous molecular markers that form intricate networks (7–10). During environmental challenges, hypothalamus regulates the activities of these cells *via* the hypothalamo-hypophyseal portal vessels to stimulate or inhibit hormonal secretion in these endocrine cells in the pituitary (6, 11–14). CRH from hypothalamus *via* CRHR controls various physiological functions for homeostasis, which is a major signaling pathway in response to different stresses (2, 5, 6).

Previously, we showed that hypoxic stress activated the HPA axis while inhibiting the hypothalamus-pituitary-thyroid (HPT) and hypothalamus-pituitary-gonadal (HPG) axes (15–17). We uncovered a biphasic response of CRHR1 mRNA signaled *via* HIF-1α and nuclear factor kappa B (NF-κB). Moreover, gestational hypoxia-induced sex-differential methylation of CRHR1 in the paraventricular nucleus (PVN) linked to anxiety-like behavior in adult offspring, and overactivation of CRHR1 and aquaporin was in cerebral cortex by hypoxia-induced brain edema (18–20). However, molecular mechanisms of CRHR1 mediated hypoxia stress response, particular concerning CRHR1 downstream targets and signaling pathways, are still far from clear. In the present study, we first determined the transcriptional regulatory networks of hormones and their receptors in the rat pituitary under hypoxic stress. Acute hypoxia (1 day) or constant hypoxia (5 days) induced distinct transcriptome changes in the pituitaries of adult male rats. We also identified the networks linking specific pituitary transcriptional factors, HIFs, and hormones and their receptors under hypoxic stress. Finally, we demonstrated the new targets of CRHR1 in rat pituitary gland under hypoxia.

## Materials and methods

### Animals

Adult male Sprague-Dawley rats weighing 140 ± 20 g were supplied by the Experimental Animal Center of Zhejiang Province, China (SCXK(Zhe)2019-0002). The rats were maintained under constant conditions (21 ± 2°C, 40–60% humidity, 12 h light/dark cycle, lights on 06:00–18:00) and fed with a standard diet and drinking water. They were housed in the lab for one week to adapt to the environment before hypoxia. All experiments were conducted in accordance with the NIH laboratory animal care guidelines. All protocols concerning animal use were approved by the Zhejiang University Animal Care and Use Committee (ZJU201304-1-01-025).

### Hypoxic stress and drug treatment

Rats in the hypoxia group were placed in a transparent and ventilated hypobaric chamber (Avic Guizhou Fenglei Aviation Armament Co., Ltd, China, FLYDWC-50-IIC) and exposed to hypobaric hypoxia of 5000 m (10.8% O_2_) for, 1 day, or 5 days (21). To avoid isolation stress, groups of 6 rats were kept in one cage under hypoxia; the average amount of group food and water intake (g or ml/rat/day) was measured after hypoxia. The normoxia group was placed in an identical chamber set at sea level (20.9% O_2_). In validation experiments, rats received injections of the CRHR1 antagonist CP-154,526 or saline (30 mg/kg, ip, donated by Pfizer, Groton, CT) (19, 21) before hypoxic stress (**Figure 5A**). After hypoxia, rats were deeply anesthetized with pentobarbital sodium (2%), decapitated trunk blood was collected, prior to remove the pituitary and brain. Samples were immediately frozen in liquid nitrogen, and stored at –80°C [n = 3 for transcriptome analysis (three groups of control, hypoxia 1 day, and hypoxia 5 days), n = 6 for the validation experiment].

### Library construction and sequencing

Total RNA was isolated and purified using TRIzol reagent (Invitrogen, Carlsbad, CA, USA) according to manufacturer’s protocol. The amount and purity of each RNA sample was quantified using NanoDrop ND-1000 (NanoDrop, Wilmington, DE, USA) with RNA concentration >50 ng/μL, OD260/280 >1.8, total RNA >1 μg. The RNA integrity was assessed by Bioanalyzer 2100 (Agilent, CA, USA) with RIN number >7.0, and confirmed by electrophoresis with denaturing agarose gel. Poly(A) RNA was purified twice from 1 μg total RNA using Dynabeads Oligo (dT) (Cat. 25-61005, Thermo Fisher, CA, USA). Then the poly(A) RNA was fragmented into small pieces using the Mg^2+^ RNA fragmentation module (Cat. E6150S, NEB, USA) at 94°C for 5–7 min. Then the cleaved RNA fragments were reverse-transcribed to synthesize cDNA by SuperScript™ II Reverse Transcriptase (Cat. 1896649, Invitrogen), which was used to synthesize U-labeled double-stranded DNA with *E. coli* DNA polymerase I (Cat. M0209, NEB), RNase H (Cat. M0297, NEB), and dUTP solution (Cat. R0133, Thermo Fisher). An A-base was then added to the blunt ends of each strand, preparing them for ligation to the indexed adapters. Each adapter contained a T-base overhang for ligating the adapter to the A-tailed fragmented DNA. Single- or dual-index adapters were ligated to the fragments, and size selection and purification were performed with AMPureXP beads. After treatment of the U-labeled second-stranded DNAs with the heat-labile UDG enzyme (NEB, Cat. M0280, USA), the ligated products were amplified with PCR by the following protocol: initial denaturation at 95°C for 3 min; 8 cycles of denaturation at 98°C for 15 s, annealing at 60°C for 15 s, and extension at 72°C for 30 s, and then final extension at 72°C for 5 min. The average insert size for the final cDNA library was 300 ± 50 bp. Finally, we performed 2×150 bp paired-end sequencing (Model PE150) on an Illumina Novaseq™ 6000** **(LC-Bio Technology Co., Ltd, Hangzhou, China) following the vendor’s recommended protocol.

### Sequencing and analyses

Cutadapt software (https://cutadapt.readthedocs.io/en/stable/,version:cutadapt-1.9) was used to remove the reads that contained adaptors. Low-quality bases and undetermined bases failed pass threshold of quality were also removed using CleanData. HISAT2 software (https://daehwankimlab.github.io/hisat2/,version:hisat2-2.0.4) was used to map reads to the rat genome. The mapped reads of each sample were assembled using StringTie (http://ccb.jhu.edu/software/stringtie/,version:stringtie-1.3.4d.Linux_x86_64) with default parameters. Then, transcriptomes from all samples were merged to reconstruct a comprehensive transcriptome using gffcompare software (http://ccb.jhu.edu/software/stringtie/gffcompare.shtml,version: gffcompare-0.9.8.Linux_x86_64). After a final transcriptome was generated, StringTie and ballgown (http://www.bioconductor.org/packages/release/bioc/html/ballgown.html) were used to estimate the expression levels of each transcript, which was assessed by calculating FPKM [total exon fragments/mapped_reads (millions) × exon length (kB)]. The differentially-expressed genes were selected with a relative mRNA expression level fold-change >2 or <0.5 and p value <0.05 using the R package edgeR (https://bioconductor.org/packages/release/bioc/html/edgeR.html) or DESeq2 (http://www.bioconductor.org/packages/release/bioc/html/DESeq2.html), and then GO and KEGG enrichment analyses of the differentially-expressed genes were conducted (22–24).

### Construction of a topographic pituitary transcription networks and a HIF-1α networks with target genes, hormones and their receptors

Based on the cell signaling pathways on NIH, the list of HIF-1α target genes (TargetScan 50), and the data from the rat pituitary transcriptome, we constructed rat pituitary specific gene transcriptome networks (receptors, hormones, and transcription factors, **Figures 3A, B**) induced by hypoxic stress. We mapped the HIF-1α, ROS, and HIF-related cancer signaling pathways (**Figure 4**) using Cytoscape software (25, 26). A network carrying selected protein interactions of targeted proteins was constructed using Cytoscape by assigning source node, target node, and combined score as edge attributes. The combined score was computed by combining the probabilities from the different evidence channels and corrected for the probability of randomly observing an interaction (25, 26).

### Predictions and analyses of transcription factors and receptors involved in hypoxia

Transcription factors and the number of transcription factors at promoters were predicted and analyzed using JASPAR software online (http://jaspar.genereg.net/) with cut-off selection for the matrix to minimize false negatives, including 11 transcription factors and 9 receptor genes (*Crhr1*: ENSRNOG00000004900, *Crhr2*: ENSRNOG00000011145, *Sstr2*: ENSRNOG00000002793, *Ghrhr*: ENSRNOG00000011808, *Gnrhr*: ENSRNOG00000002011, *Trhr*: ENSRNOG00000005048, *Prlr*: ENSRNOG00000057557, *Drd1*: ENSRNOG00000023688, *Mc4r*: ENSRNOG00000018692), producing hormone factors (CRH, PRL, GH, TSH, and LH/FSH) (*Crh*: ENSRNOG00000012703; Prl: ENSRNOG00000017374; *Gh1*: ENSRNOG00000011207; *Tshb*: ENSRNOG00000016793; *Lhb*: ENSRNOG00000047040; *Fshb*: ENSRNOG00000004898) in the pituitary (27). All gene sequences were obtained from the database https://asia.ensembl.org/index.html.

### Quantitative real-time PCR

Total RNA was extracted using TRIzol reagent (Life Technologies). RNA (500 ng) was reverse-transcribed into cDNA using the Transcript™ RT enzyme mix (TransGenBiotech, China) and then stored at –20°C. cDNA was subjected to PCR amplification using TB Green Premix Ex Taq™ (Takara, RR420A). The primer sequences for qRT-PCR were listed in **Supplementary Table 1**. β-actin was amplified as an endogenous control, and all samples and negative controls were prepared in duplicate wells of a 384-well plate and analyzed using the PRISM7900HT real-time PCR system (Applied Biosystems, Foster City, CA, USA). The cycle threshold (CT value) was used to calculate the relative amount of mRNA. The CT value of each target was normalized by subtraction of the CT value of β-actin.

### Immunofluorescence of c-Fos

Immunofluorescence analysis was carried out on cryostate sections of rat pituitary glands as previously reported (19). Briefly, after postfixation and washes, rat pituitary glands were placed in a cryoprotective solution of PBS/30% sucrose for 12 h. Continuous coronal sections (rostral to caudal, 16 µm) were obtained using a cryostate and stored at -80 C°. For each rat (n = 6/per group), three slides (front, middle and back) were selected from individual pituitary gland for staining. A primary antibody of c-Fos (1:1000, Synaptic Systems, 226003) and a secondary antibody that was conjugated to Alexa Fluor 546 (1:1000, Invitrogen, A11035) were used for immunofluorescence detection of c-Fos. Nucleus was counterstained with DAPI. As a negative control, PBS was used instead of the primary antibody. Images were captured at magnification (40×) using a fluorescence microscope (VS120-S6-W, Olympus, Japan). The number of c-Fos-positive (c-Fos^+^) cells was manually counted by a blind observer using CellSens Dimension software (Olympus, Japan). Per rat, six pituitary sections were counted, for each section, 4 different regions of one section were selected, and the c-Fos positive cell number were counted and averaged.

### Assays for corticosterone

Trunk blood (2 mL) was collected at the same time after stress, and the plasma levels of corticosterone were measured using a commercial ELISA kit for rats (500561, Cayman Chemical Co.). The detection threshold was 30 pg/mL. The within-assay and between-assays coefficients of variation were 3.8% and 5.6%, respectively. The antibody cross-reacted 100% with corticosterone, 11% with 11-dehydrocorticosterone, 7% with 11-deoxycorticosterone, and <0.5% with other steroid hormones. Repeated samples from individual rats were analyzed within the same assay.

### Statistical analysis

All statistical analyses used GraphPad (Prism 6) software. Data are presented as the mean ± SEM. Statistical significance compared with the control was determined with a two-tailed, unpaired Student’s t test. Comparisons among multiple variables were determined with one-way ANOVA followed by the Fisher least significance difference test. We generated the transcriptome from three adult male rat pituitaries. Body weight, food and water intake, and qRT-PCR for pituitary glands and plasma corticosterone used six rats. P <0.05 was considered significant.

## Results

### Hypoxia decreases body weight and intake of food and water

**Figure 1:**
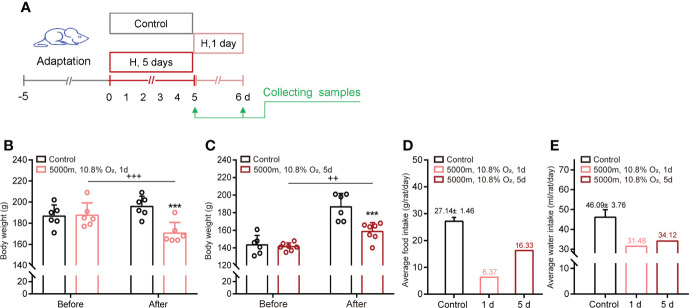
Hypoxia decreases body weight and food/water intake in adult male rats. **(A)** Schematic of the study design. **(B, C)** Body weight before and after hypoxia for 1 day or 5 days. **(D, E)** Average food and water intake during normoxia and hypoxia for 1 day or 5 days. ***P <0.001 vs control; ^++^P <0.01, ^+++^P <0.001 before *vs* after stress. Mean ± SEM, n = 6.

Exposure to hypoxia (5000 m, 10.8% O_2_) for 1 day or 5 days decreased the weight and intake of food and water in adult rats (**Figures 1A–E**). Constant hypoxia exposure (5 days) inhibited the intake every day, but especially on day one. Subsequently, food and water intake and body weight gain gradually recovered, but remained below the initial levels.

### Hypoxic stress changes gene expressions in adult rat pituitary

**Figure 2:**
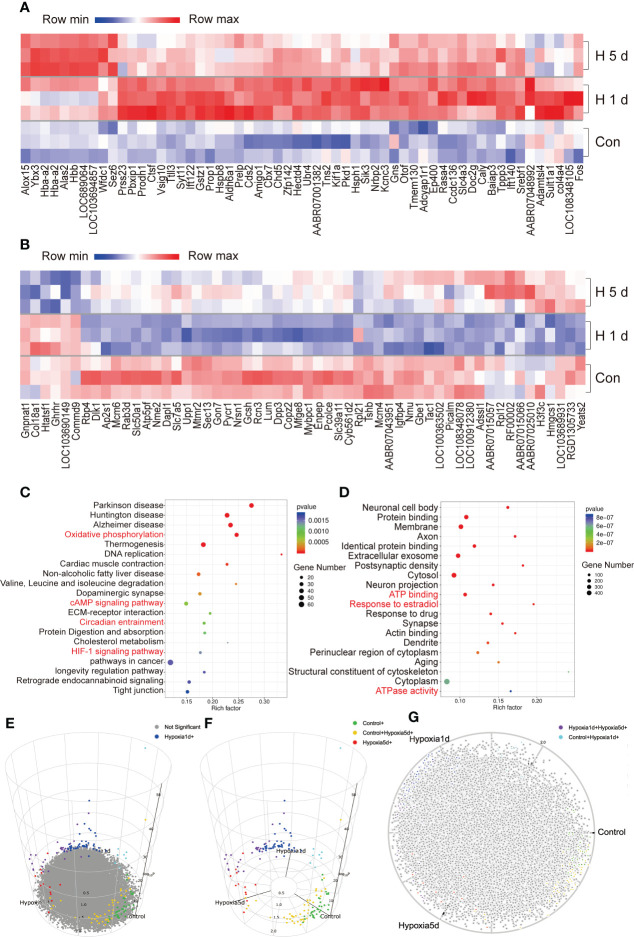
Overview of the transcriptome of adult male rat pituitary during hypoxic stress. **(A, B)** Genes up-regulated or down-regulated during hypoxic stress. **(C, D)** Top 20 KEGG Pathway and GO annotations enriched with DEGs during hypoxic stress. **(E–G)** 3D volcano maps of pituitary transcriptome from rat after hypoxic stress, the different color indicated the control, and hypoxia of 1 d and 5 days groups, the 3D map can be accessed online.

Hypoxic stress significantly altered the expression levels of various genes in the adult rat pituitaries (**Figures 2A, B**, **Supplementary Figure 1**). In both acute (1 day exposure) and constant hypoxic (5 days exposure) groups, 357 differentially-expressed genes (DEGs) were identified to undergo modification (as defined by fold-change ≥2 or ≤0.5 and p <0.05): 190 were upregulated and 167 were downregulated compared to control (control vs 1 day hypoxia, control vs 5 days hypoxia, respectively, **Supplementary Tables 2, 3**). In the acute hypoxia group, 255 genes were specifically differentially expressed (vs control), and in the continuous hypoxia group, 157 genes were specifically differentially expressed (vs control) (**Supplementary Tables 2, 3**). Nine DEGs were found to be modified in both hypoxia groups, also with different levels of modifications, which included calbindin 2 (Calb2), neuromedin U (Nmu), heat shock protein family B (Hspb7), villin 1 (Vil1), vasoactive intestinal peptide (Vip), beta-carotene oxygenase 1 (Bco1), galanin and GMAP prepropeptide (Gal), hemoglobin subunit beta (Hbb), and vomeronasal 2 receptor 44 (Vom2r44) (**Supplementary Figure 1** and **Tables 2, 3**). GO analysis of DEGs showed the enrichment for biological process, cellular component, and molecular function (**Supplementary Figure 1**). A cellular activity marker, *Fos*, was among the most strongly upregulated genes, and the expression of *Prop1*, heat shock protein h1 (*Hsph1)*, and heat shock protein b8 (*Hspb8)* also dramatically increased (**Figure 2A**). Many genes were involved in HIF-1 signaling (18 genes), oxidative phosphorylation (34 genes), cAMP signaling (31 genes), longevity regulation (16 genes), circadian entrainment (18 genes), and tight junction (25 genes) (**Figure 2C**), as well as in protein binding (246 genes), membrane (302 genes), cytosol (325 genes), cytoplasm (452 genes), ATP binding (164 genes), ATPase activity (35 genes), and response to estradiol (36 genes) (**Figure 2D**). Conversely, the expression of *Tshb*, *Ghrhr*, and *Igfbp4* decreased in pituitary cells after hypoxia (**Figure 2B**). We constructed a 3D volcano map with top and side views of the pituitary transcriptome from rats during acute and constant hypoxic stress (**Figures 2E–G**), and listed the genes upregulated and downregulated during hypoxic stress for 1 day or 5 days vs control (**Supplemental Table 2**), and 1 day vs 5 days (**Supplementary Table 3**).

### Impact of hypoxia on transcription of hormonal factors and their receptors in the pituitary

The six populations (corticotrophs, somatotrophs, lactotrophs, gonadotrophs, melanotrophs and thyrotrophs) of pituitary cells were identified using previously-published gene markers (7–10, 14). Acute or constant hypoxia induced diverse changes in gene expression in these groups. Genes normally expressed in somatotrophs (*Gh*, *Ghrhr*, *Sstr5*, *Sstr2*) were all downregulated following acute or constant hypoxia (**Supplementary Figure 2**).

**Figure 3:**
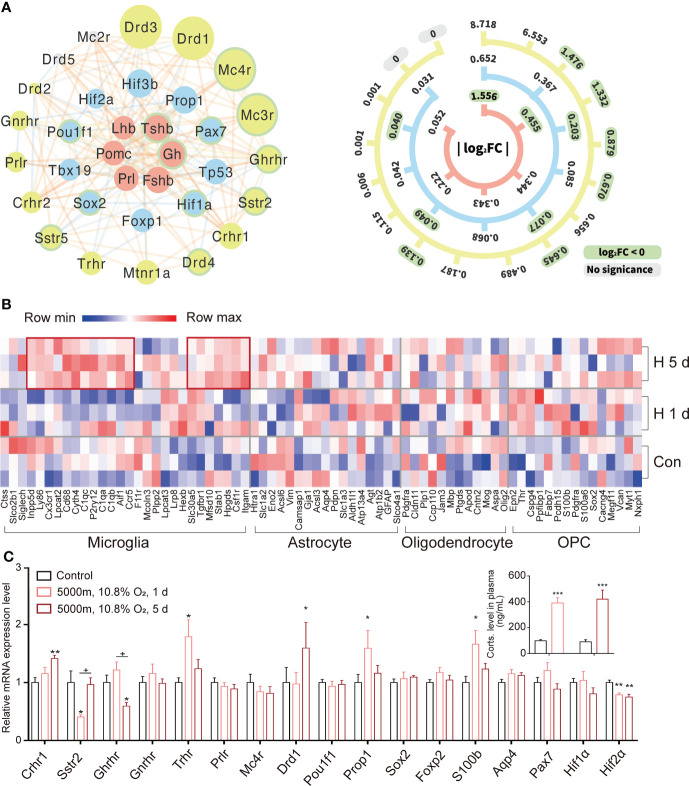
Transcriptional expression and networks for representative hormones and their receptors in rat pituitary during hypoxic stress. **(A)** Transcriptional networks of classical hormones (red), their receptors (yellow), and transcription factors (blue) during hypoxia stress response in rat pituitary. The size of each circle is proportional to the fold-change in gene expression under constant hypoxia. The scale bar on the right indicates exact fold-changes. Down-regulated genes are highlighted with green borders. Lines connect genes with potential functional associations predicted by STRING. **(B)** Heatmap for genes expressed in subgroups of glial cells during hypoxia. **(C)** Expression changes of representative receptors and transcriptional factors validated by qRT-PCR in rat pituitaries (n = 6) during 1 or 5 days (d) hypoxic stress. Insert: changes of plasma corticosterone (Corts) following 1 or 5 days (d) hypoxia exposure in comparison to control (black bar). Values are the mean ± SEM, n = 6,*P<0.05, **P<0.01,***P<0.001 vs control;+P<0.05, hypoxia 1 d vs hypoxia 5 d.

Based on the public databases of special markers (http://bis.zju.edu.cn/MCA/index.html; http://biocc.hrbmu.edu.cn/CellMarker) and the transcriptomes (9, 10) of murine pituitary, we constructed a three-ring-network of representative hormones, receptors, and transcription factors with *Hif* and *Tp53* in rat pituitaries induced by hypoxia (**Figure 3A**). At the center of the network, we found stronger expressions of *Prl*, *Gh*, *Pomc*, and *Tshb*. While at the outermost ring, we found the lower-level expressions of receptors such as *Crhr1*, *Crhr2*, *Prlr* (PRL receptor), *Drd*1 (dopamine receptor), *Gnrhr* (GnRH receptor), *Sstr2* (somatostatin receptor 2), *Ghrhr* (GHRH receptor), *Trhr* (TRH receptor), and *Mc4r* (melanocortin receptor 4) (28). Special transcriptional factors, pituitary transcription factors, HIFs, and *Tp53* were the relay center between the two groups that regulated the transcription *via* the binding sites of the promoters of target genes (**Figure 3A** and **Supplementary Figure 3**). Uninterrupted hypoxia (5 days exposure) upregulated the expression of several marker genes for microglia (68% active) (**Figure 3B**); however, the majority of other glial cell markers were not significantly expressed in the adult rat pituitary during normoxia or hypoxia. Additionally, there are some activated gene makers under hypoxia for stem cells and glial cells (**Supplementary Figure 4**), as previously reported (29, 30). We selected a few representative genes and used qRT-PCR to further validate RNAseq results, found that 5 days of constant hypoxia, increased the expression of *Crhr1* and *Drd1*, and decreased *Ghrhr* and *Hif-2α* (**Figure 3C**). As expected, hypoxia (1 day or 5 days) activated HPA axis and significantly increased plasma corticosterone (**Figure 3C** and inset). Our results showed that a dynamic regulatory expression pattern could be found in the pituitary transcriptional micro-networks (**Figure 3A**).

### Hypoxic stress activates the HIF-1α signal pathway and target genes

**Figure 4:**
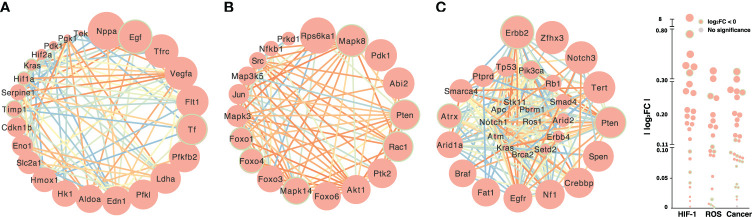
Transcriptional expression of the HIF-1 **(A)**, ROS **(B)** and HIF-related cancer **(C)** signal pathways under hypoxic stress. The sizes of circles are proportional to the difference in gene expression between normoxia and constant hypoxia. Downregulated genes are highlighted with green borders. Lines connect genes with potential functional associations predicted by STRING, and the color of each line represents the strength of the association quantified by the combined score generated by STRING.

HIFs are transcriptional factors and oxygen sensors for homeostasis that are activated by various physiological and pathophysiological processes (31–33). Both acute and continuous hypoxic stress stimulated the expression of *Hif*, HIF target genes and signaling pathways: natriuretic peptide type A (*Nppa*), vascular endothelial growth factor A (*Vegfa*), transferrin receptors (*Tfrc*), aldolase (*Aldoa*), enolase 1 (*Eno1*), lactate dehydrogenase A (*Ldha*), and phosphoglycerate kinase 1 (*Pgk1*) (**Figures 4A** and **2C**). *Hif-1α* has not been reported to be implicated in the ROS and HIF-related cancer signal pathways (**Figures 4B, C**). The gene signatures of HIF-1α, ROS, and HIF-related cancer signals under control *versus* uninterrupted hypoxia (**Figure 4**) and control *versus* acute hypoxia (**Supplementary Figure 5**) were distinct. Ribosomal protein S6 kinase A1 (*Rps6ka1*), pyruvate dehydrogenase kinase 1 *(Pdk1)*, abscisic acid insensitive 2 *(Abi2)*, and AKT serine/threonine kinase 1 (*Akt1*) were upregulated in the ROS signal pathway, while the HIF-related cancer signal converged on *Notch3*, the SWT/SNF (switch/sucrose non-fermentable) chromatin remodeling gene (*Smarca4*), zinc finger homeobox 3 (*Zfhx3*), and telomerase reverse transcriptase (*Tert*). Comparing the signal pathways showed different patterns between normoxia and acute or nonstop hypoxia in the heatmaps (**Supplementary Figure 5**). *Zfhx3* and *Notch3* in the HIF-related cancer signal pathway were upregulated under hypoxia, while phosphatase, tensin (*Pten*), and receptor tyrosine-protein kinase (*Errb2)* were downregulated after continuous hypoxia. Downregulated *Pten* occurred in both the ROS and HIF-related cancer signal pathways (**Figures 4B, C**); the expression of some HIF target genes, such as *Kras* (Kirsten rat sarcoma 2 viral oncogene homolog, GTPase KRas oncogene), was also downregulated in the HIF-related cancer signal and HIF pathway. Also, there were strong connections in the HIF-related cancer signal pathway under hypoxia (**Figure 4C**). We constructed a network for HIF-1 with target genes, and hormones and their receptors by STRING, where the significant link were color coded to indicate the strength of associations quantified by the combined score generated by STRING (**Supplementary Figures 6A, B**). There were strong connections and potential crosstalk between the two networks of HIF and hormone factors at the transcriptional level.

### CRHR1 mediates the regulation of certain hormones and their receptors in rat pituitary during hypoxia

**Figure 5:**
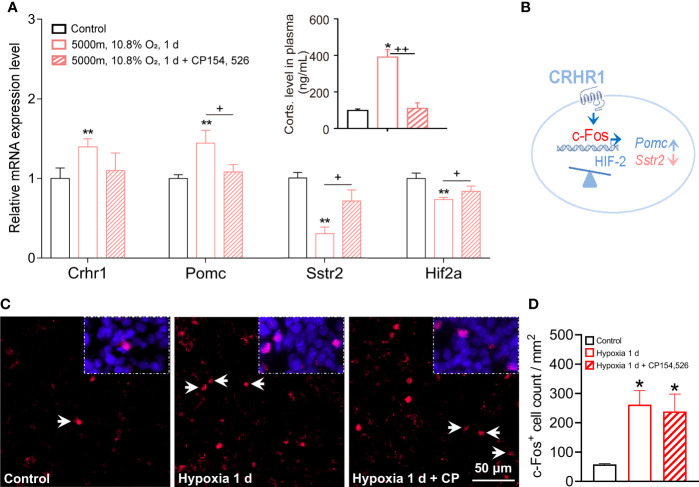
CRHR1 mediated hypoxia induced changes of hormones, receptors, and pituitary transcription factors in adult rat pituitaries. **(A)** CRHR1 mediated the transcriptional regulation of several hypoxia-responsive genes including Pomc, Sstr2, and Hif2α. Effects of CRHR1 were blocked by CP-154,526, a potent and specific antagonist of CRHR1. **(B)** Proposed transcriptional regulation of the hypoxia-responsive genes via CRHR1. **(C, D)** Representative immunofluorescent images and quantification of c-Fos positive (c-Fos^+^) cells number in anterior pituitary. Values are the mean ± SEM, n = 6, *P <0.05, **P <0.01, vs control; ^+^P <0.05, ^++^P <0.01, hypoxia vs hypoxia + CP154,526 in **(A)**; *P <0.05, vs control in **(D)**.

Because of *Crhr1* was significantly upregulated during hypoxia, we hypothesized that CRHR1 mediated changes of certain hypoxia genes such as transcriptional factors, pituitary hormones, and their receptors that were critical for hypoxia responses in rat pituitaries. Rats were treated with hypoxia in combination with or without a specific CRHR1 antagonist (CP154,526). Consistent with results of our previous studies (18, 19), exposure to hypoxia significantly increased plasma levels of corticosterone, while a CRHR1 antagonist (CP154, 526) blocked this hypoxia-induced stress response (**Figure 5A** and inset). Exposure to hypoxia for 1 day at a dose of 10.8% O_2_ (or severe hypoxia 8% O_2_, 8 h) significantly increased transcripts of *Crhr1* and *Pomc* (**Figure 5A**, **Supplementary Figures 7A, B**), but decreased expression of *Sstr2* and *Hif-2α* (**Figure 5A**). Importantly, blocking of CRHR1 effects by a CRHR1 specific antagonist (CP154,526), i.e., increase of *Pomc* and decrease of *Sstr2* and *Hif-2α* (**Figure 5A**), suggest that CRHR1 mediates the regulation of gene expression of these hypoxia genes. One day hypoxia exposure (10.8% O_2_) also significantly increased expressions of *Creb*, *Ghrhr*, *Prl*, *Lhb*, and *S100b*, and decreased *Tshb* (**Supplementary Figure 7C**); however, these changes could not be blocked by this CRHR1 antagonist (CP154,526), which suggest existence of alternative signaling pathways (**Supplementary Figure 7C**). We proposed a working model for hypoxia induced gene expression mediated *via* CRHR1 (**Figure 5B**).

Using bioinformatics, we determined the distribution and number of transcription factors of the HIF family (ARNT, ARNT2, ARNTL, and HIF-1α) and *Prop1*, that bind to the promoters of hormones *(POMC, Gh, Prl, Tsh, Lh*, and *Fsh)* (**Supplementary Table 4**) and their receptors (**Supplementary Table 5**) that are important for the regulation of transcription and chromatin accessibility during stress. Large numbers of ARNT, HIF-1α, and SOX2 (mean = 34, 11, and 11) binding sites were found in the promoter regions of targeted receptors, such as the *Gh, Sstr2* and *Mc4r* promoters. This result suggested that the transcription factors, HIFs play important roles in regulating the transcription of pituitary hormones and their receptors during hypoxic stress.

Meanwhile, we observed a significant increase of c-Fos^+^ cells that represented the increase of secretory activities of anterior pituitary cells in response to hypoxia (**Figures 5C, D**). Lesser c-Fos^+^ signals and cell count were seen in anterior pituitary following the treatment of CRHR1 antagonist CP154,526; however, this decrease was not significant (**Figure 5D**). Interestingly, genes associated with the inflammatory response were also active in the pituitary. Acute hypoxia downregulated the expression of *Tp53*, interleukin 1 receptor type 2 (*Il1r2*), interleukin 2 receptor subunit beta (*Il2rb*), and Toll-like receptor 8 (*Tlr8*) (**Supplementary Figure 8**), while upregulated the expression of interleukin 6 receptor (*Il6r*), interleukin 6 signal transducer (*Il6st*), and interleukin 17 receptor D *(Il17rd*) (**Supplementary Figure 8**). In addition hypoxia upregulated the transcription levels of chemokine C-X-C motif chemokine ligand 12 (*CXC12*; vs hypoxia 5 d) and cAMP-responsive binding protein 3-like 2 (*Creb3l2*; vs hypoxia 1 d), in accordance with upregulated *Creb* transcription (**Supplementary Figure 7C**) and an active cAMP signaling pathway during hypoxic stress (**Figure 2C**) as previously reported (34).

## Discussion

### Acute and continuous hypoxia impact multiple gene populations in the HIF, ROS and HIF-related cancer signalling pathways

Genes from classical pituitary endocrine cell populations have been detected and identified, consistent with reports using single cells, genomes, and transcriptomes from mouse, rat, and human pituitary (7–10, 14), and are also supported by proteomes in the human pituitary (35–37). In the present study, more than 100 genes were involved in the positive and negative regulation of transcription by RNA polymerase II, along with signaling pathways, including the HIF pathway, ATP binding and ATPase activity, the response to estradiol (**Figures 2C, D**), oxidative phosphorylation, and the cAMP signaling pathway in response to hypoxia. There are numerous of O_2_-dependent reaction in the body, HIFs are cellular sensor for adaptation and response to the variation of oxygen level. HIFs were not involved in NOS and the HIF-related cancer signaling pathway during hypoxia for 5 days. ROS production in pituitary might serve as a signaling mechanism that promotes cell proliferation, differentiation and growth. The HIF signaling pathway was distinct from ROS and HIF-related cancer signaling under hypoxic stress (**Figure 4**); 26 genes were upregulated in the HIF-related cancer signaling pathway after 1 day of hypoxia, but only 5 of these remained upregulated after 5 days of hypoxia (**Supplementary Figure 5**).

We found upregulated expression of *Fos*, *Kifla*, and *Prop1*, and downregulated expression of *Tshb*, *Ghrhr*, and *Igfbp4* (**Figures 2A, B**) under continuous hypoxic condition. *Fos* and *Prop1* are known marker genes for active cells and specific pituitary transcription factors (9, 10, 14); these results were validated by qRT-PCR of representative genes (**Figure 3C** and **Supplementary Figure 7**) and immunofluorescence of c-Fos^+^ (**Figures 5C, D**). Evidence from our previous reports also supports the idea that hypoxia stimulates CRH and PRL release in the short term, but inhibits GH, Gnrh, and TRH release in the long term (15–20). In photoperiod stress, transient hypoxia, and prenatal alcohol stress, similar transcriptional changes have been reported in fetal sheep and rat pituitary (7, 38, 39). Together, hypoxia dynamically changes the pituitary cellular and molecular functions *via* multidimensional networks of transcription, translation, hormone production and secretion.

### The genes for both hormones and their receptors are essential in brain-pituitary-target cells

In the current adult rat pituitary transcriptome, genes have been identified for corticotrophs/melanotrophs (*Pomc* cells) (8, 40), somatotrophs, lactotrophs, gonadotrophs, and thyrotrophs. We added the receptor’s gene of these cell populations *Crhr1/Crhr2* and *Mc4r*, *Ghrhr* and *Sstr2/Sstr5*, *Prlr* and *Drd1/Drd2/Drd3/Drd4*, *Gnrhr* and *Trhr* to a new pituitary transcription-network with the oxygen-sensor HIFs to reveal the mechanisms of transcriptional regulation under hypoxic stress (**Figures 3A** and **5**). To validate, we confirmed that hormones, receptors, and transcriptional factors coregulate the pituitary functions. Under hypoxia, *Prl*, *Lhb* upregulated, whereas *Tshb* downregulated; *Crhr1*, *Trhr*, and *Drd1* increased, while *Crhr2*, *Sstr2*, and *Ghrhr* decreased in the pituitary. Tachykinin-1(*Tac1)* is expressed in thyrotrophs and somatotrophs in rat pituitary single-cell transcritptome (10). *Tac1* gene products, namely substance P (SP) and neurokinin A (NKA), *Tac1* also decreased under hypoxia of 1 d and 5 d (**Figure 2B**). In a clinical report, the DEGs of gonadotroph tumors were downregulated, while those of somatotroph tumors were mainly upregulated (41). *Sstr2*, anti-Sstr2 antibody drugs and Sst-image have been developed for diagnosis and therapy in pituitary and neuroendocrine tumors (42, 43); signaling-mediated plasma membrane resurfacing of Sstr2 can fine-tune pituitary hormone release (44).

In accordance with the inhibited GH-insulin-like growth factor 1 axis, HPG axis, and HPT axis under continuous hypoxia in previous studies from our lab (17, 18, 20), hypoxia for 1 and 5 days downregulated the expression of somatotroph cell markers, and decreased the mRNA levels of *Ghrhr* and *Sstr2* (**Figures 3C**, **5A** and **Supplementary Figures 2, 7C**), as well as increased *Trhr* and decreased *Tshb* (**Figure 3C**, **Supplementary Figures 7C**). For the HPA axis, we used stress models with different levels of acute (10.8% or 8% O_2_ for 8 h) and continuous hypoxia (10.8% O_2_ for 5 days): acute hypoxia induced a rapid *Crhr1* transcription and an HPA axis response (**Supplementary Figure 7**).

*Pomc* is expressed in both melanotrophs and corticotrophs (9, 40). Using qRT-PCR, we found that acute hypoxia (8% O_2_, 8 h or 10.8% O_2_, 1 d) significantly increased *Pomc* expression (**Figure 5A**, **Supplementary Figure 7B**), and CRHR1 antagonist blocked hypoxia induced *Pomc* expression (**Figure 5A**) and ~10.0% c-Fos^+^ signal (**Figures 5C, D**). CRHR1 is a member of heptahelical G protein-coupled receptors in the family B (secretin-like hormone receptors, subfamily B1), higher expression in anterior and intermediate lobs of the pituitary, and mainly localized in ACTH expressing corticotropes, where CRHR1 stimulation triggers the secretion of ACTH into circulation (1). Therefore, the dynamic regulatory of CRHR1 transcription in HPA axis seems involve in the phosphorylation, desensitization/resensitization, and recycling of CRHR1 during successive exposure to stressful stimuli and resilience. Under hypoxia challenge, a cellular activity marker, c-Fos and oxygen sensor of *Hif2* mediates HPA axis function *via* CRHR1 as proposed working model (**Figure 5B**). A recent report has demonstrated that pioneer *Pax7* specifies melanotroph cells, and chromatin opening requires both *Pax7* and *Tbx19* (Tpit, T-box19, T-box) cooperation; the pioneer factor opens closed chromatin, and the non-pioneer provides chromatin opening (39). *Pomc, Crhr1*, and *Tbx19* are marker genes for corticotrophs (9), *Pomc* and *Pax7* for melanotrophs (9), and proprotein convertase subtilisin/kexin type 2 (*Pcsk2*) is also for melanotrophs (40). In the present study, melanocortin receptor (*Mc3r*) and *Mc4r* were added for melanotrophs (28), even though the expression levels were lower. We included *Pomc* in the two subgroups of corticotrophs and melanotrophs with their individual receptors. Data from single cell RNA seq reveals that corticotrophs, melanotrophs, gonadotrophs, thyrotrophs, somatotrophs, lactotrophs, and progenitor cells share *Pomc* genes in the mouse pituitary, with higher expression in corticotrophs and melanotrophs (40). This suggests that the individual receptors of classic hormones are important to identify specific functional axes of the pituitary. *Pomc, c-Fos* and *Crhr1* might be good markers for the HPA’s stress response, because we demonstrated that hypoxia activated the HPA axis *via* the type 1, but not the type 2 CRH receptor during acute and continuous hypoxia. Remarkably, a CRHR1 antagonist blocked the corticosterone responses to hypoxia (**Figure 5A** & inset). Another report also supports: continuous application of a GnRH receptor agonist blocks *Fshb* expression and depletes the LH secretory pool in the rat pituitary, while transcription of *Gnrhr* is involved in this process (45). Two distinctive *Pomc* promoters were identified to change gene expression in Cushing disease, demythelation of the 2^nd^ *Pomc* promoter is associated with aggressive pituitary ACTH-secreting tumors (46). This indicated the regulatory transcription function of receptors in brain-pituitary-target axis during continuous stress. Not only does the ligand bind to the receptor protein at the membrane, but the transcription factors also bind to the promoters of target receptor genes in the nucleus. This suggests that transcription of the receptors for specific hormones also contributes to regulation of the transcription rate of hormone in the pituitary by regulating the actions of releasing and/or inhibiting hormones from the PVN of the hypothalamus *via* a feedback loop. Lack of blocking by CRHR1 antagonist in the c-Fos protein signals (**Figures 5C, D**) and transcription of *Creb*, *Ghrhr*, *Prl*, *Lhb* and *S100b*, but fully inhibition of hypoxia-induced high levels of corticosterone suggest this specific CRHR1 antagonist only blocks CRHR1 action in ACTH cells.

In addition, there is also crosstalk among different receptors, such as Drd1, Sstr2/5, and Mc4r and the melanotonin receptor (Mrnt1), which coordinates and mediates PRL, GH, and LH or FSH release as well as the circadian rhythm (6, 37, 47). Studies of the network of somatotropes and lactotropes have revealed a role for cell organization in gene regulation and receptor co-localization, and juxtacrine/paracrine and autocrine action in pituitary cells (40, 48). Chimeric compounds of the Sstr2/5/Drd2 system have been explored in receptor crosstalk mechanisms in the pituitary. Based on previous reports, there is also an acknowledged drug combination of a GnRH antagonist and GH in central precocious puberty (49, 50). Therefore, the pituitary has a heterogeneous and complex cellular organization of hormone cells with their receptors as well as different transcriptional factors, and has multiple foci for brain-target cells (HPA, HPT, and GH axes) *via* their receptors and transcriptional factors, as well as integrating and regulating the pituitary micro-network (**Figure 3A**).

HIFs and TP53 in pituitary are regulators for cell proliferation and apoptosis, HIFs are the master regulators of oxygen homeostasis during hypoxia. A balance of HIF-1α to HIF-2α signaling is required in order to adapt cells to prolonged hypoxia (33) and targeting genes transcription. The pituitary gland has a reservoir of stem cells that ensure its lifelong plasticity (3, 36, 51). We found downregulation of genes in non-endocrine proliferating Pou1f1 (pituitary transcription factor) in all pituitary cells (**Supplementary Figure 4**) (4, 36, 37) and upregulation of genes in stem cells and glial cells (microglia, astrocytes, oligodendrocytes, and OPCs) even at low expression levels during hypoxia, so hypoxic treatment for stem cell research and developmental plasticity is need for a deeper understanding (52). Most gene markers for microglia in the pituitary are active under hypoxia for 5 days, and active microglia in the frontal cortex contribute to hypoxia-induced edema (19). OPC markers in the rat and mouse pituitary remain ambiguous, based on the ranking of different marker genes (14, 29). However, transplanted OPCs (F3.olig2 cells) restore rat neurobehavioral functions after hypoxia-ischemia-lipopolysaccharide injection by preventing axonal demyelination (53). This is supported by the coupling of HIF signaling intrinsic to OPCs, postnatal white matter angiogenesis, and the onset of myelination in the mammalian forebrain (30). Hypoxic stress is also a good research model for the involvement of stem cells and OPCs in cell development, proliferation, and differentiation (30, 34, 54). Hypoxic insult is the proposed mechanism of pituitary dysfunction after traumatic brain injury in humans with severe anemia, hypotension, or hypoxia (4, 30, 55, 56). *Tp53* has been reported to be involved in normal somatotroph apoptosis (57, 58), but *Tp53* mutation does not occur in pituitary adenomas. It is noteworthy that significantly enhanced expression of *Prop1* and *Tp53* in the pituitary under hypoxia may contribute to cell proliferation and apoptosis during development, puberty, and lactation, rather than as a HIF-related cancer signal.

### Hypoxia switches the HIF signal and target genes through transcriptional regulation and transcription factors

The upregulated expression of genes was associated with chromatin modeling through chromatin, the accessibility mechanisms of serine threonine kinase 11 *(Stk11)*, *Smarca4*, and AT-rich interactive domain (*Arid1a, BAF250a)*, which impact the gene transcription process. Transcriptional factors, HIFs can bind to a specific binding site, hypoxia response element (HRE) in the promoter of the target gene (31–33), therefore, we propose that the HIFs are candidates for pioneer or non-pioneer factors that might be involved in the regulation of transcription during pituitary development and homeostasis during physiological and psychological stress (37, 40, 54, 59, 60). We demonstrated that hypoxic stress increased *Crhr1* and *Trhr* and decreased *Sstr2* (**Figures 3C**, **5A**), while increasing *Hif-1α* in acute hypoxia (**Supplemental Figure 7A**) and decreasing *Hif-2α* in continuous hypoxia for 5 days. HIFs and *Propl* bind to the promoters of the target genes of hormones and/or their receptors to dynamically regulate transcriptional expression in the pituitary (topographic) network (**Figure 3A** and **Supplementary Figure 6**). This is consistent with the previous report that HIF-1α and NF-κB positively control transcription while c-Jun/AP-1 negatively regulates transcription in rat pituitary and AtT20 cells, because eight HIF-1 binding sites, one HAS (HIF ancillary sequence), four NF-κB, and five Jun/AP-1 sites are present in the rat *Crhr1* gene promoter (61). Moreover, we have shown previously that demethylation of the *Crhr1* promoter in the PVN is also involved in male offspring anxiety behavior as a result of gestational hypoxia (62). Another group reported that Hif-1α activates protein kinase A by repressing RIIβ subunit transcription in human GH-secreting pituitary tumor cells; HIF-1α represses the transcription of the gene encoding RIIβ (PRKAR2B) (34). Here, we found that HIFs as transcription factors occurred at the promoters of hormones and their receptors respectively **(Supplemental Tables 4, 5**); this means that HIFs can bind to the promoters of *Sstr2, Sstr 5, Ghrhr, Gnrhr, Trhr, Prlr, Drd1*, and *Mc4r* to regulate their transcription pattern during hypoxic stress, such as sleep apnea of hypoxia, chronic obstructive pulmonary disease (33, 63). The balance between HIF-1α and HIF-2α controls cardiovascular homeostasis. Chronic continuous hypoxia induced-HIF-1α and HIF-2α results in pulmonary hypertension, whereas chronic intermittent hypoxia induces HIF-1α but inhibits HIF-2α, generating systemic hypertension. Hif-1α^+/-^ and Hif-2α^+/-^ mice are both protected from hypoxic pulmonary hypertension. Besides, the Tibetan HIF-2α variants associated with an absence of an erythrocytotic response to high altitude hypoxia might encode the reduced HIF-2a function. Endothelial HIF-2a in adult mice, but not Hif-1α, is required to maintain airway microvascular structure and function (33), and HIF-2α antagonist is renal cell cancer target (64). In the oxygen-induced retinopathy mouse model, rapid but transient accumulation of Hif-1α, but delayed and sustained accumulation of Hif-2α. Staggered Hif expression was corroborated in hypoxic adult mouse retinal explants but not in human retinal organoids (65). In our study, CRHR1 antagonist can abolish the decreased transcription of *Sstr2* and *Hif-2α*, it seems that this small molecule can re-balance of transcription of oxygen-sensor Hif-1*α* and Hif-2*α* during hypoxia stress.

Involvement of HIF and HRE has also been reported in the fetal sheep pituitary cell transcriptome and human GH3 cells (7, 34, 54). Decreased *Arnt* expression has been reported in pituitary adenomas from aryl hydrocarbon receptor-interacting protein *(Aip* ^+/–^) mice (66). In addition, we assessed the expression of a set of specific transcription factors (*Foxp2, Sox2, Pax7*, and *Pou1f1*) in adult rat pituitary during continuous hypoxia, and unexpectedly found no change except for enhanced *Prop1 in vivo*. Those indicated our hypoxia model in health adult rat pituitary only mimic physiological stress, but are not a pathological cancer model yet, and therefore HIFs as oxygen sensors are dispensable in the HIF-related caner signal pathway (**Figure 4**). We suggest that transcription factors HIFs and pituitary transcriptional factors co-regulate the gene transcription at the promoters of the respective hormones and receptors *via* the binding sites/cis-acting elements.

Hypoxia activated the expression of chemokine and cytokine receptors for immune response. Previously, we reported that hypoxia activated cytokine release from the cortex and circulating leukocytes, and increased the cytokine level of IL-1β, IL-6, and TNF-α in the circulation (19, 67), while NF-κB is involved in *Crhr1* transcription and brain edema (19, 61). Here, there were significant changes in chemokine and cytokine receptors in the rat pituitary (**Supplementary Figure 8**): acute hypoxia for 1 day induced upregulation of *Il6r* and *Il17rd* with *Nfkb1* (also in the ROS signaling pathway, **Figure 4B**) and downregulation of *Il2rb, Il1r2*, and *Tlr8*. This is consistent with the findings that PDTC (NF-κB inhibitor) blocks the hypoxia-induced *Crhr1* mRNA expression in the pituitary and corticosterone in the circulation (19, 61). Moreover, PDTC blocks lipopolysaccharide-induced water permeability in cultured astrocytes (67). The human blood transcriptome also shows enhanced immune and inflammatory responses after exposure to a high-altitude plateau (5300 m, 3 days) in humans with acute mountain sickness (68). These results suggest the dynamic regulation of cytokine gene expression in the inflammatory response during hypoxic stress. Chemokinergic regulation of the CXCL12-CXCR4 signaling pathway interfaces with the classical endocrine pathway in hypoxia-induced GH production in pituitary adenoma cells (69, 70). This indicates that both cytokine receptors and transcription factors contribute to the inflammatory responses in the pituitary during hypoxic stress.

## Conclusion

Dynamic transcriptional regulation maintains the physiological homeostasis *via* transcription factors, c-Fos with HIFs, hormones, and receptors in the pituitary during hypoxic challenge. We constructed a new functional topographic network of transcription factors, hormones, and receptors. c-Fos, HIFs, and pituitary transcription factors might co-regulate the transcription activities of hormones and their receptors *via* binding to the promoters. CRHR1 mediates the transcriptional regulation of *Pomc*, *Sstr2 via* c-Fos under hypoxia stress. This study provides insights into understanding the mechanisms of temporal-transcriptional regulation of specific pituitary receptors, c-Fos with HIFs for hormones and their receptors in health and disease.

## Data availability statement

The original contributions presented in the study are publicly available. This data can be found here: NCBI, PRJNA804340.

## Ethics statement

The animal study was reviewed and approved by Zhejiang University Animal Care and Use Committee.

## Author contributions

TW and XC drafted the manuscript. XC and JD conceived of and supervised the project. BS, MC, XX, and FX contributed to primer design and animal care. YZ, ML, TL, and JG contributed to figure and manuscript editing. FX, MZ, and QL conducted immunofluorescence and ELISA. XL and DZ contributed to bioinformatics analysis. All authors contributed to the article and approved the submitted version.

## Funding

This research was supported by the National Natural Science Foundation of China (81930054 and 32120103007), the National Basic Research Program (973) of the Ministry of Science and Technology of China (2012CB518200), the Non-profit Central Research Institute Fund of the Chinese Academy of Medical Science (2018PT31041), and a 111 project (B13026).

## Acknowledgments

We dedicate this paper to the memory of Ji-Zeng Du, our dear supervisor, who passed away on June 30, 2017, while this work was in progress. Prof. Du contributed greatly to understanding the mechanisms of brain-endocrine-immune network function during high altitude hypoxia. We thank Prof. G. Leng from the University of Edinburgh for critical comments and Prof. I.C. Bruce at Peking University for editing the paper, and the technical support by the Core Facilities, Zhejiang University School of Medicine.

## Conflict of interest

The authors declare that the research was conducted in the absence of any commercial or financial relationships that could be construed as a potential conflict of interest.

## Publisher’s note

All claims expressed in this article are solely those of the authors and do not necessarily represent those of their affiliated organizations, or those of the publisher, the editors and the reviewers. Any product that may be evaluated in this article, or claim that may be made by its manufacturer, is not guaranteed or endorsed by the publisher.
