# Supplementary material for: CRHR1 mediates the transcriptional expression of pituitary hormones and their receptors under hypoxia
Source: Front Endocrinol (Lausanne). 2022 Sep 2;13:893238. doi: 10.3389/fendo.2022.893238 (PMC9487150; doi:10.3389/fendo.2022.893238)
Supplement: Supplementary file 1 [file DataSheet_1.docx]

**Supplemental Information**

Figures 1-8 and Tables 1-5

**Supplemental Fig. 1.** Numbers of genes involved in biological process, cellular component, and molecular function during hypoxic stress by GO analysis. The Venn diagram shows the numbers of displayed differentially-expressed genes (DEGs) in the pituitary after hypoxic stress for 1 day and 5 days.

**Supplemental Table 1.** The primer sequences for Q-RT-PCR.

| Primer | Sequence 5' → 3' | Primer | Sequence 5' → 3' |
| --- | --- | --- | --- |
| Crfr1 | (F): TCCACTACATCTGAGACCATTCAGTACA | Sox2 | (F) GCTGGGAGAAAGAAGAGGAG |
|  | (R): TCCTGCCACCGGCGCCACCTCTTCCGGA |  | (R) ATCTGGCGGAGAATAGTTGG |
| Sstr2 | (F): TACTTCGTGGTGTGCGTGGTG | Foxp2 | (F) TATGGAGCAGCCCTTAATGC |
|  | (R): CTTGGCGTAGCGGAGGATGAC |  | (R) GGTTACTTAGCAAAGGCAAACTG |
| Ghrhr | (F): AGGGGCTGTGAAAAGGGACTG | Pax7 | (F) GCGCGGTCCCCAGGATGATG |
|  | (R): CAGTGTCCTCAAAAGCCAGTT |  | (R) ACGCGGAGCTGACGGGAGAT |
| Gnrhr | (F): GTGGTGATTAGCCTGGATCG | Hif1a | (F) ACCATGCCCCAGATTCAAGA |
|  | (R): ATAACTGTGGTCCCGCAAAG |  | (R) ATCGCTGTCCACATCAAAGC |
| Trhr | (F): ACAGAACGGTGGAAATACCG | Hif2α  (Epas1) | (F) CTGGCCCATGTCTACCATCT |
|  | (R): TCTGTGGCTTGGTGCAGTAG |  | (R) CCAAGGCGTGTTAGATCTGC |
| Prlr | (F) CTGGGCAGTGGCTTTGAAG | Arntl  (Bmal1) | (F) TGCCACTGACTACCAAGAAAGT |
|  | (R) CCAAGGCACTCAGCAGCTCT |  | (R) ATTTTGTCCCGACGCCTCTT |
| Mc4r | (F): CTTGCACAGTATCGGGCGTTCT | Pomc | (F) CCATAGACGTGTGGAGCTGG |
|  | (R): GTTCTTGACTCCGCAGGGCATA |  | (R) AGGGCTGTTCATCTCCGTTG |
| Drd1 | (F) CAAAGCAGCCTTCATCCTGA | S100b | (F) ACCCACATCTGGCAGAATGAG |
|  | (R) ATGGCATACGTCCTGCTCAA |  | (R) AGCCATGACCTTTCGCATTAG |
| Pou1f1 | (F) ACTCAGGGTGTGGTCTGGAAACTT | Aqp4 | (F) TGGTCCTCATCTCCCTCTGCTT |
|  | (R) ATGTCCACAGCGACAGGACTTCAT |  | (R) TGAACCGTGGTGACTCCCAATCC |
| Prop1 | (F) TCCTGACATCTGGGTTCGAG | β-actin | (F) AGCCATGTACGTAGCCATCC |
|  | (R) GGAGTAGTGACCGCTCTTGC |  | (R) CTCTCAGCTGTGGTGGTGAA |
| Creb | (F)accagcagagtggagatgct | Gh1 | (F) ccttgtccagtctgtttgcc |
|  | (R)gggctaatgtggcaatctgt |  | (R)aatggaatagcgctgtccct |
| Pomc | (F) TTCAAGAGGGAGCTGGAAGG  (R) TGATGGCGTTCTTGAAGAGC | Tshb | (F)gtgggcaagtgtcatcgttt  (R) agacatcctgagagagtgcg |
| Prl | (F)aggaacaaaacaagcggctt  (R) gcaggcaccgaatgttgtta | Lhb | (F) tagtctcctttcctgtggcc  (R) ggggaaggtcacaggtcatt |
| Fshb | (F) gtcgatccagctttgcatcc  (R) gtttggtctagctgggtcct |  |  |

**Supplemental Table 2.** Differentially-expressed genes in rat pituitary between hypoxia and control.

| H1 d  *vs* Con | H5 d  *vs* Con | Gene name list (Total number of genes) |
| --- | --- | --- |
| ↑ | ↑ | Mapt, Cnksr2, Fos, Hemgn, Epb42, Ddi2, Kcna2, Alox15, Slc4a1, Amer3, Col6a4, AABR07066416, Lyc2, LOC102553828, AABR07006889, AABR07019086, Hbb (17) |
| ↑ | — | Prodh1, Rasl11a, Irs3, Gap43, Eva1c, Hpse, Corin, Wfikkn2, Cxcr3, Cby3, Prop1, Tdrd5, RGD1308775, Vit, Tmem74, Best3, Erich5, Reg3b, Reg3a, Fbxo32, Adhfe1, Atp8a2, Jhy, Ttll3, Mapk15, Rspo4, Prlhr, Serpina3c, LOC299282, Mpp4, Nmb, Zdhhc22, Dlgap2, Fabp3, Wnt4, Tll2, Nova2, Ccn1, Gipr, Marveld3, Gnmt, Eya4, Pla2g2f, Itih3, Lipg, Pik3ip1, Rhbg, Msln, Cyp1a1, AABR07026957, Galnt15, Npas4, AABR07005821, Slc22a18, Trim72, Galp, Trim50, Plec, LOC680875, Dmrta1, LOC103689978, Disp3, Pigc, Tfap2e, Pnma8a, Tnni3k, AABR07048992, Mlc1, Shc4, Catip, Tmem229a, RGD1565355, RGD1560775, Arc, AABR07033887, AABR07016141, Cited4, Fosb, Clic5, Gns, LOC100360647, LOC108348105, Nek5, D430019H16Rik, LOC108348105, Rack1, Nat8l, Lrg1, Hspa1b, AABR07021536, AABR07064502, AABR07070312, AABR07014974, AABR07031489, Kcna6, AABR07043453, AC096792, Apoa4, AABR07052897, Myom1, Insrr, AABR07051308, Adcy1, Astn2, AABR07043407, Acot12, AABR07064878 (107) |
| — | ↑ | Alas2, Prf1, Arntl2, LOC100362453, Klf1, Atf3, Il1b, Ngfr, Phospho1, Slc6a11, Rbm38, Guca2b, RGD1310507, Car3, Radx, Lgals5, Ksr1, Cd52, Cdh17, Kel, Mab21l3, Acbd7, Emilin3, Fam210a, LOC308990, Cartpt, Apoc1, Ccdc24, Ahsp, Lingo4, Hfm1, Cxcl13, Pf4, Hbq1a, Hba-a2, AABR07027015, Insyn2, Rarres1, Hba-a3, AABR07015066, LOC689064, Hba-a2, AABR07002546, Pidd1, Robo4, AABR07063425, AABR07064716, RF00002, AABR07010468, LOC102554500, LOC100910710, Rpph1, AABR07015081, AABR07027872, AABR07068030, RF00004, RF00030, LOC100909609, Ldlrad3, RF00100, Gata5, Asphd2, AABR07015042, LOC103694857, Grtp1, Cers1 (66) |
| — | ↓ | Pappa2, Avp, AY172581, Calca, AY172581, Casp12, AABR07012097, AABR07060872, Rgn, RGD1565131, Obp2a, AY172581, Cfap161, Cidea, AABR07072853, Tmem202, AABR07036087, Cfap53, Cdh19, LOC103690149, Ifit1, NEWGENE_1306714, Gnpnat1, Trpm6, AABR07004112, Mocos, Mchr1, Tnrc6b, AABR07000398, Klhdc7a, Selenbp1, Itprip, Tbx6, LOC100360491, Nfs1, AABR07038029 (36) |
| ↓ | — | LOC103689931, Mybpc1, Pycr1, Ces1c, Nrsn1, Fgl2, Dapl1, Enpep, Slc39a11, Pcolce, Myh9, B4galt6, Tac1, AABR07054319, Krt79, Mcm3, Sstr2, Ceacam16, Racgap1, Calcb, Scn10a, Dctd, Ccnt1, Krt71, Scnn1g, Knl1, Il1r2, Cdc6, Dtl, AABR07044001, Arhgap9, Scn4a, Cacna2d4, Bcl3, AABR07028989, Exo1, Galnt6, Bpifa5, Sstr3, Stac2, Cdt1, Mypn, Krt76, Gjb2, LOC100912599, LOC100359951, Pvalb, Tph1, Galnt5, Slc22a12, Fuom, Mms22l, AABR07072264, E2f8, Hist2h4a, AABR07017902, Slc25a45, Nppc, Ak5, Ncapg, Smim24, Cxcl9, Trip13, Clec2l, Ccer2, Rpl30, Myo7a, RF00017, Mcm10, Pcdhb6, AABR07019383, AABR07022162, LOC690507, LOC100911725, Kcnk5, Mis18a, Cabp2, AC117065, Col17a1, Gins1, Ltbp2, AABR07000452, Tlr8, Fignl1, AABR07030156, S100a4, AABR07044404, AABR07066435, LOC108351370, Aunip, AABR07032724, Tmem45al, C1qtnf7 (93) |
| ↓ | ↓ | Nmu, Polq, Capn8, Srpx2, AABR07072759, Clcnkb, Vom2r44, Bco1, Melk, Gal, Vil1, Fcrl2, Tshb, Calb2, Pla2g2e, Vip, Ecel1, AABR07051507, Ctdspl2, Hspb7, AY172581, Ndufa1, Ccdc122, Bves, LOC102549173, AC117058, B9d1, Gckr, LOC100364500, Mep1b, Rpl21, LOC100910143, RT1-CE10, LOC100910990, Hmg1l1, AABR07014855, Cftr, Myh15 (38) |

Note: ↑ up-regulation (190), ↓ down-regulation (167), — no significant difference; Differentially-expressed genes (DEGs) defined by fold-change ≥2 or ≤0.5 and p <0.05.

**Supplemental Table 3.** Differentially-expressed genes in rat pituitary between hypoxia for 1 day and 5 days.

| H5 d  *vs* H1 d | Gene name list (Total number of genes) |
| --- | --- |
| ↑ | LOC103689931, B4galt6, Calb2, LOC103692170, AABR07015057, Fgl2, LOC100360791, Hba-a3, Ccnt1, LOC689064, Hspb7, Hba-a2, RF00017, AABR07015066, LOC103694857, Hbb, Ces1c, Tac1, Galnt14, AABR07027015, Vil1, Cxcl9, Alas2, RF00004, RF00002, Calcb, Nmu, Krt79, LOC100911485, Cd52, Myh9, RF00100, Racgap1, Smim24, AABR07072264, Lingo4, AABR07041600, LOC100909609, Cartpt, Vip, Nppc, Mis18a, Galnt6, Bco1, Slc22a3, LOC103692471, Tph1, Guca2b, AABR07063425, Krt23, LOC100912599, AABR07056464, Car3, AABR07041109, Shisa8, AABR07060610, AABR07010468, Tnni1, Ak5, Ctse, AABR07015042, Acap1, Cd300le, LOC100910270, LOC102547056, LOC103690141, 1700092M07Rik, Il2rb, Ccl2, Lypd8, AABR07015081, Vnn1, AABR07064724, Bpifa5, RF00017, Stac2, Col14a1, Tlr8, AABR07063425, Ncan, Mos, Il21r, Cers1, AABR07066020, C1qtnf7, LOC102554500, Rpph1, Acbd7, C1qtnf12, Cdh17, AABR07064716, AABR07066435, Myo7a, Prss12, AABR07062533, Prf1, AABR07001512, AABR07035722, RF00030, LOC103692570, Gal, Cacng3, Elk4, Gbp6, Slc6a13, Galns (106) |
| ↓ | LOC103690149, Ifit1, Plec, Gnpnat1, Adhfe1, Nfs1, Apoa4, Mlc1, LOC108348105, Prodh1, Selenbp1, Zfp7, NEWGENE_1306714, AABR07005821, LOC108348105, Nmb, Cldn1, Sult1a1, AABR07048992, Ghrhr, RGD1308564, Insrr, Olr1418, AABR07000398, Tbx6, AC096792, RGD1560775, LOC689757, Rsph4a, LOC103689978, LOC100360491, Corin, Zdhhc22, Galp, Map7d3, AABR07028009, AC120712, Rasef, LOC100361636, AABR07036087, Tnrc6b, Ntsr2, AABR07020879, Serpina3c, Npas4, Lrg1, Ccdc170, Hsd17b13, D430019H16Rik, AABR07043564, AABR07066693, Kcnk10, Cyp1a1, Trim72, Rasl11a, AC119762, Tspan4, Fezf1, AABR07038029, Dlgap2, Itprip, Depp1, Atp4a, Calhm1, Fbxo32, Reg3b, Arc, Egr3, Vom2r44, Klhdc7a, Coch, Ccn1, Atoh7, Reg3a, LOC100911027, Tmem116 (76) |

Note: ↑ up-regulation (106), ↓ down-regulation (76); DEGs defined by fold-change ≥2 or ≤0.5 and p <0.05.


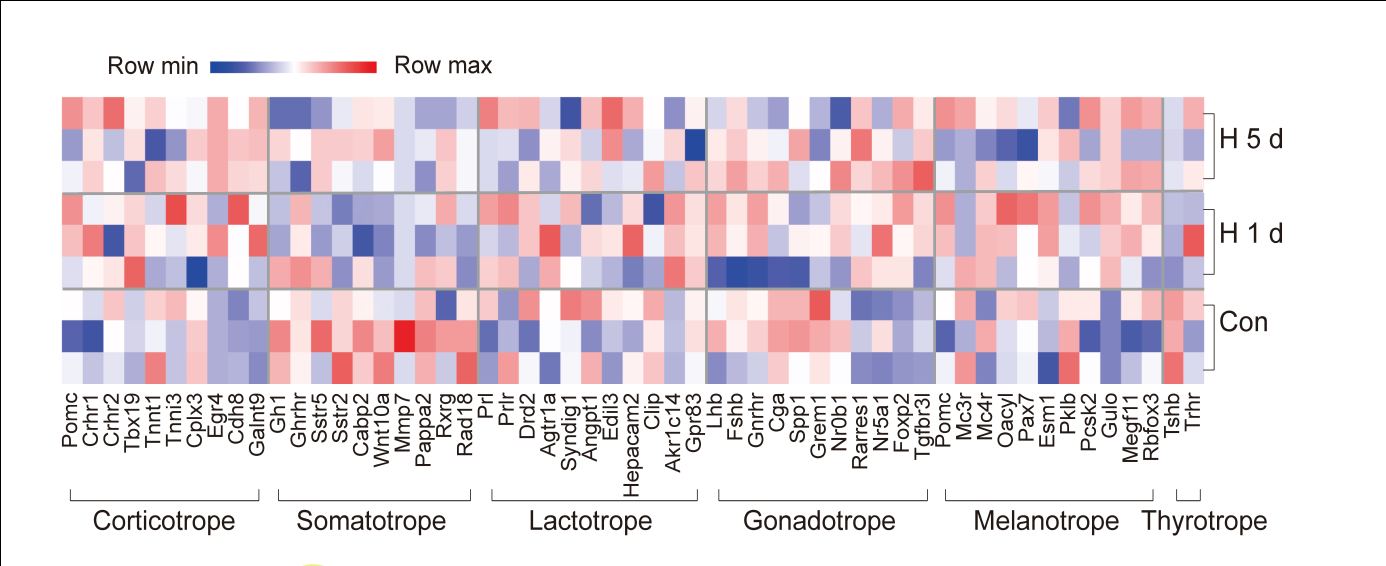


**Supplemental Fig.2.** Gene expression of classical hormones during hypoxia.

**Supplemental Fig. 3.** Hypoxia-pituitary network for control and hypoxia 1 d (see Fig. 3A for 5 days). The size of a circle is proportional to the fold-change of gene expression levels during hypoxic stress (scale bar in right side based on fold change), and down-regulated genes are highlighted with green borders. The links in the network represent the various potential functional associations quantified by the combined score generated by STRING.

**Supplemental Fig. 4.** Heatmap of gene expression for proliferating Poulf1 cells, stem cells, and glial cells during hypoxic stress.

**Supplemental Fig. 5.** Heatmaps and network for the Hif and ROS signals, and HIF-related cancer signaling pathway during hypoxia for 1 day and 5 days. Hypoxia upregulates the gene expression in different signal pathways (A1-C1). The three rectangles in C1 indicate much stronger expression at 5 day (C_1_) or 1 days (C_2_, C_3_) of hypoxia (*vs* control). The network represents the various potential functional associations predicted by STRING (A2, B2, C2, control *vs* 1 d hypoxia). The size of a circle is proportional to the fold-change of gene expression levels under hypoxia for 1 d, downregulated genes are highlighted with green borders (DEGs defined by fold-change ≥2 or ≤0.5 and p <0.05).

**Supplemental Fig. 6.** The network for Hif1 with target genes and their receptors and hormones during hypoxic stress. (*A, B*) Comparison of gene expression in the network of Hif target genes with pituitary hormones and receptors under hypoxia in rat pituitary (*A*, control *vs* 1 d in top; *B*, control *vs* 5 d below); blue, hormone factors with pituitary transcription factors Prop1 and Pou1f1. The size of circles indicates the fold-change of gene expression levels under hypoxia for 1 day and 5 days; down-regulated genes are highlighted with green borders. Gene class 1, 2 is HIF and target genes, gene class 3 presents the hormone factors, gene class 4 is their receptors.

**A B**


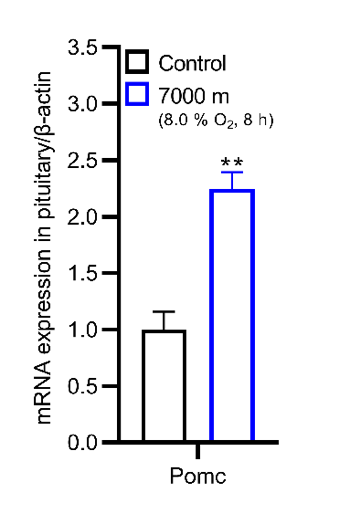


**C**


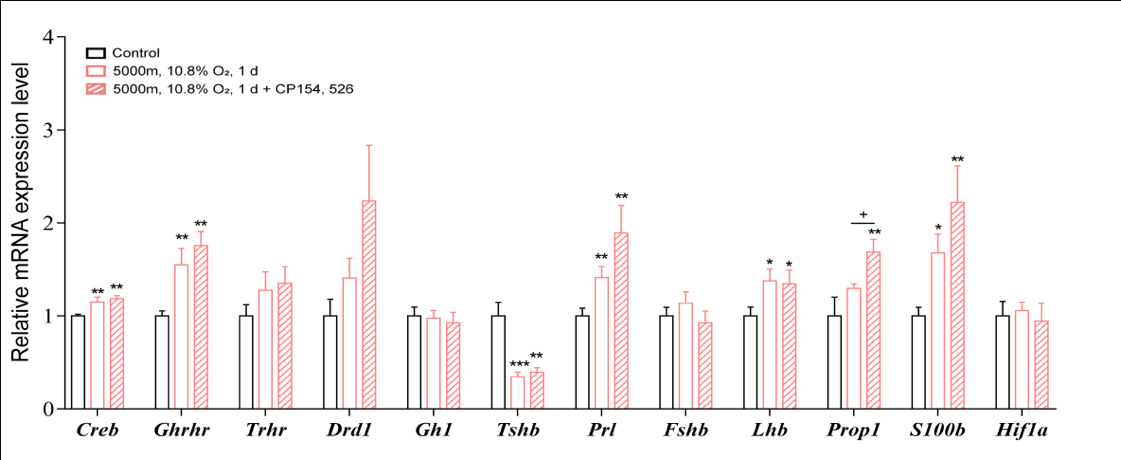


**Supplemental Fig. 7.** Expression of *Pomc*, *Creb*, *Hif*, hormones and their receptors in the rat pituitary under hypoxia. Data are presented as the mean ±SEM, n = 6. **P* <0.05, ***P* <0.01, ****P* <0.001 *vs* control, ^+^P <0.05, ^++^P <0.01, ^+++^P <0.001 *vs* 2000 m hypoxia group; ^#^P <0.05, ^##^P <0.01, ^###^P <0.001 *vs* 5000 m hypoxia group in (*A*); **P <0.01, *vs* control in (*B*); *P <0.05, **P <0.01, ***P <0.001 *vs* control; ^+^P <0.05, hypoxia *vs* hypoxia + CP 154,526 in (*C*).

**Supplemental Table 4.** Number of predicted transcription factors at promoters (–3000 to –1 bp)

of classical hormones in the pituitary.

|  |  | Arnt | Arnt2 | Arntl | Hif1α | Foxp2 | Nfkb1 | Nfkb2 | Pax7 | Prop1 | Sox2 | Tp53 |
| --- | --- | --- | --- | --- | --- | --- | --- | --- | --- | --- | --- | --- |
| *Prl* | + | 62 | 0 | 2 | 15 | 14 | 19 | 3 | 3 | 6 | 20 | 1 |
|  | - | 26 | 1 | 2 | 4 | 15 | 9 | 3 | 5 | 8 | 20 | 2 |
| *Gh1* | + | 78 | 4 | 3 | 18 | 8 | 7 | 1 | 0 | 0 | 27 | 2 |
|  | - | 39 | 4 | 4 | 21 | 16 | 7 | 1 | 0 | 0 | 17 | 4 |
| *Tshb* | + | 50 | 2 | 1 | 6 | 1 | 7 | 0 | 0 | 0 | 8 | 0 |
|  | - | 41 | 2 | 1 | 13 | 4 | 6 | 0 | 0 | 0 | 7 | 0 |
| *Lhb* | + | 57 | 1 | 0 | 22 | 8 | 19 | 4 | 0 | 0 | 15 | 0 |
|  | - | 29 | 1 | 0 | 10 | 5 | 17 | 4 | 0 | 0 | 8 | 0 |
| *Fshb* | + | 33 | 3 | 2 | 17 | 13 | 2 | 0 | 4 | 6 | 13 | 0 |
|  | - | 26 | 3 | 9 | 4 | 10 | 4 | 0 | 4 | 4 | 24 | 0 |
| *Pomc* | + | 23 | 4 | 3 | 2 | 11 | 15 | 4 | 0 | 3 | 26 | 0 |
|  | - | 41 | 3 | 3 | 17 | 10 | 19 | 3 | 1 | 3 | 29 | 0 |
| Average | + | 56.00 | 2.00 | 1.60 | 15.60 | 8.8 | 10.80 | 1.60 | 1.40 | 2.40 | 16.60 | 0.60 |
|  | - | 32.23 | 2.20 | 3.20 | 10.40 | 10.00 | 8.60 | 1.60 | 1.80 | 2.40 | 15.20 | 1.20 |
| SEM | + | 6.02 | 0.58 | 0.42 | 2.17 | 1.89 | 2.83 | 0.66 | 0.71 | 1.20 | 2.64 | 0.33 |
|  | - | 2.65 | 0.48 | 1.30 | 2.59 | 2.02 | 1.84 | 0.66 | 0.91 | 1.31 | 2.73 | 0.65 |

**Supplemental Table 5.** Numbers of predicted transcription factors at promoters (–3000 to –1 bp) for

the respective receptors in the pituitary.

|  |  | Arnt | Arnt2 | Arntl | Hif1α | Foxp2 | Nfkb1 | Nfkb2 | Pax7 | Prop1 | Sox2 | Tp53 |
| --- | --- | --- | --- | --- | --- | --- | --- | --- | --- | --- | --- | --- |
| *Crfr1* | + | 21 | 0 | 0 | 10 | 1 | 10 | 2 | 0 | 0 | 8 | 0 |
|  | - | 42 | 0 | 0 | 13 | 2 | 19 | 1 | 0 | 0 | 10 | 0 |
| *Crfr2* | + | 54 | 1 | 0 | 6 | 5 | 6 | 1 | 1 | 3 | 16 | 1 |
|  | - | 24 | 0 | 0 | 7 | 4 | 9 | 1 | 1 | 1 | 17 | 2 |
| *Sstr2* | + | 53 | 9 | 5 | 29 | 7 | 9 | 0 | 3 | 1 | 8 | 4 |
|  | - | 74 | 20 | 6 | 27 | 2 | 6 | 0 | 3 | 2 | 11 | 0 |
| *Ghrhr* | + | 18 | 4 | 3 | 11 | 4 | 23 | 5 | 1 | 1 | 2 | 19 |
|  | - | 18 | 5 | 4 | 10 | 11 | 19 | 5 | 2 | 1 | 2 | 19 |
| *Gnrhr* | + | 18 | 3 | 2 | 8 | 6 | 6 | 1 | 5 | 10 | 1 | 18 |
|  | - | 18 | 4 | 3 | 7 | 10 | 8 | 1 | 4 | 13 | 0 | 24 |
| *Trhr* | + | 34 | 4 | 5 | 17 | 10 | 4 | 0 | 4 | 5 | 24 | 0 |
|  | - | 34 | 4 | 2 | 9 | 13 | 2 | 0 | 4 | 3 | 34 | 0 |
| *Prlr* | + | 33 | 6 | 5 | 13 | 13 | 5 | 0 | 1 | 1 | 19 | 1 |
|  | - | 43 | 6 | 5 | 10 | 4 | 10 | 0 | 1 | 1 | 28 | 2 |
| *Drd1* | + | 45 | 3 | 3 | 8 | 2 | 7 | 0 | 0 | 1 | 3 | 0 |
|  | - | 4 | 2 | 1 | 2 | 3 | 5 | 0 | 0 | 1 | 1 | 0 |
| *Mc4r* | + | 23 | 4 | 3 | 9 | 13 | 7 | 1 | 5 | 8 | 14 | 0 |
|  | - | 63 | 5 | 3 | 10 | 10 | 14 | 1 | 4 | 5 | 16 | 0 |
| Average | + | 33.22 | 3.78 | 2.89 | 12.33 | 6.78 | 8.56 | 1.11 | 2.22 | 3.33 | 10.56 | 4.78 |
|  | - | 35.56 | 5.11 | 2.67 | 10.56 | 6.56 | 10.22 | 1.00 | 2.11 | 3.00 | 13.22 | 5.22 |
| SEM | + | 4.55 | 0.83 | 0.62 | 2.21 | 1.39 | 1.80 | 0.51 | 0.64 | 1.12 | 2.55 | 2.48 |
|  | - | 7.09 | 1.88 | 0.67 | 2.16 | 1.37 | 1.88 | 0.50 | 0.53 | 1.27 | 3.75 | 2.94 |


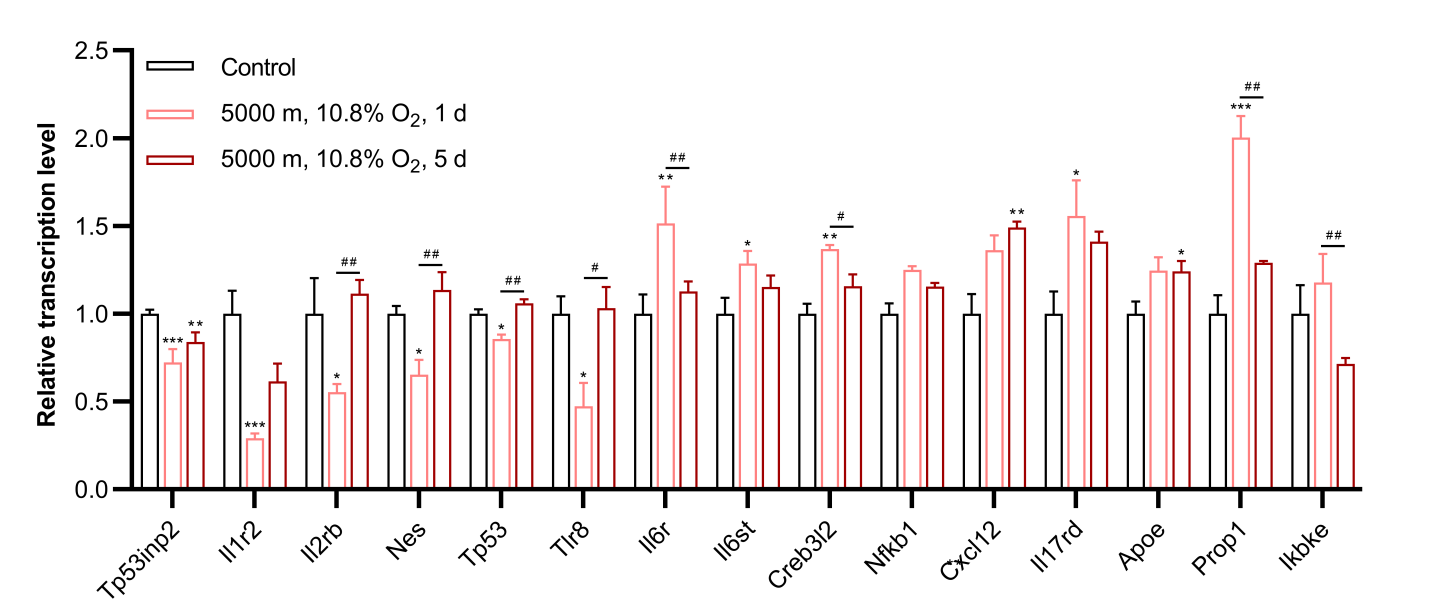


**Supplemental Fig. 8.** Inflammatory response genes in rat pituitary during hypoxic stress. **P* <0.05, ***P* <0.01, ****P* <0.001 *vs* control; ^#^P<0.05, ^##^P<0.01, for 1 d *vs* 5 d hypoxia. Data are presented as the mean ±SEM, n = 3.
